# Eosinophilic Fasciitis of Wrists with a Positive Prayer Sign

**DOI:** 10.7759/cureus.6581

**Published:** 2020-01-07

**Authors:** Xia Li, Zhen Tian, Fang Kong, Yi Zhao, Xiaoxia Li

**Affiliations:** 1 Allergy and Immunology, Xuanwu Hospital, Beijing, CHN; 2 Rheumatology, Xuanwu Hospital, Beijing, CHN

**Keywords:** prayer sign, eosinophilia, eosinophilic fasciitis

## Abstract

Eosinophilic fasciitis (EF) is an uncommon disease of unknown etiology and is characterized by inflammation and thickening of the muscular fascia and subcutaneous tissue. The patients often have peripheral eosinophilia, increased erythrocyte sedimentation rate, and hypergammaglobulinemia. In EF, the skin of the hands and feet are generally spared. Herein we present a case of EF of the wrists with a positive prayer sign and good response to corticosteroid and methotrexate. It is important to emphasize that a positive prayer sign might be the first symptom of EF. The combination regimen of systemic corticosteroids and methotrexate is recommended.

## Introduction

Eosinophilic fasciitis (EF), first described by Shulman in 1974, is characterized by erythema, edema, and induration of the extremities [1]. Symmetrical and distal lesions mainly in the forearms and legs classically appear after trauma or physical effort. Dermatological examination shows edema, skin induration with a “peau d‘orange” appearance, and venous furrowing (groove sign), but Raynaud’s phenomenon is unusual. In EF, the skin of the hands and feet are generally spared [2]. Herein we present a case of EF limited to the wrists, showing a positive prayer sign. Informed written consent was obtained from the patient for publication of this case report and accompanying images. Magnetic resonance imaging (MRI) helped guide the diagnosis and enabled us to start treatment early, which showed a good remission rate in eight months.

## Case presentation

A 34-year-old Chinese man presented with a six-month history of pain and swelling of the left knee, induration of both forearms, and limitation of dorsiflexion of the wrists for four months. Gradually, he also developed pain and swelling of the right ankle. Four months ago, he presented with symmetric induration in both forearms, which made him unable to dorsiflex the wrists, but there was no pain. He denied paresthesia and numbness in both hands. His work involved lifting heavy objects in the recent 10 years. He denied having suffered any trauma or being exposed to toxins.

**Figure 1:**
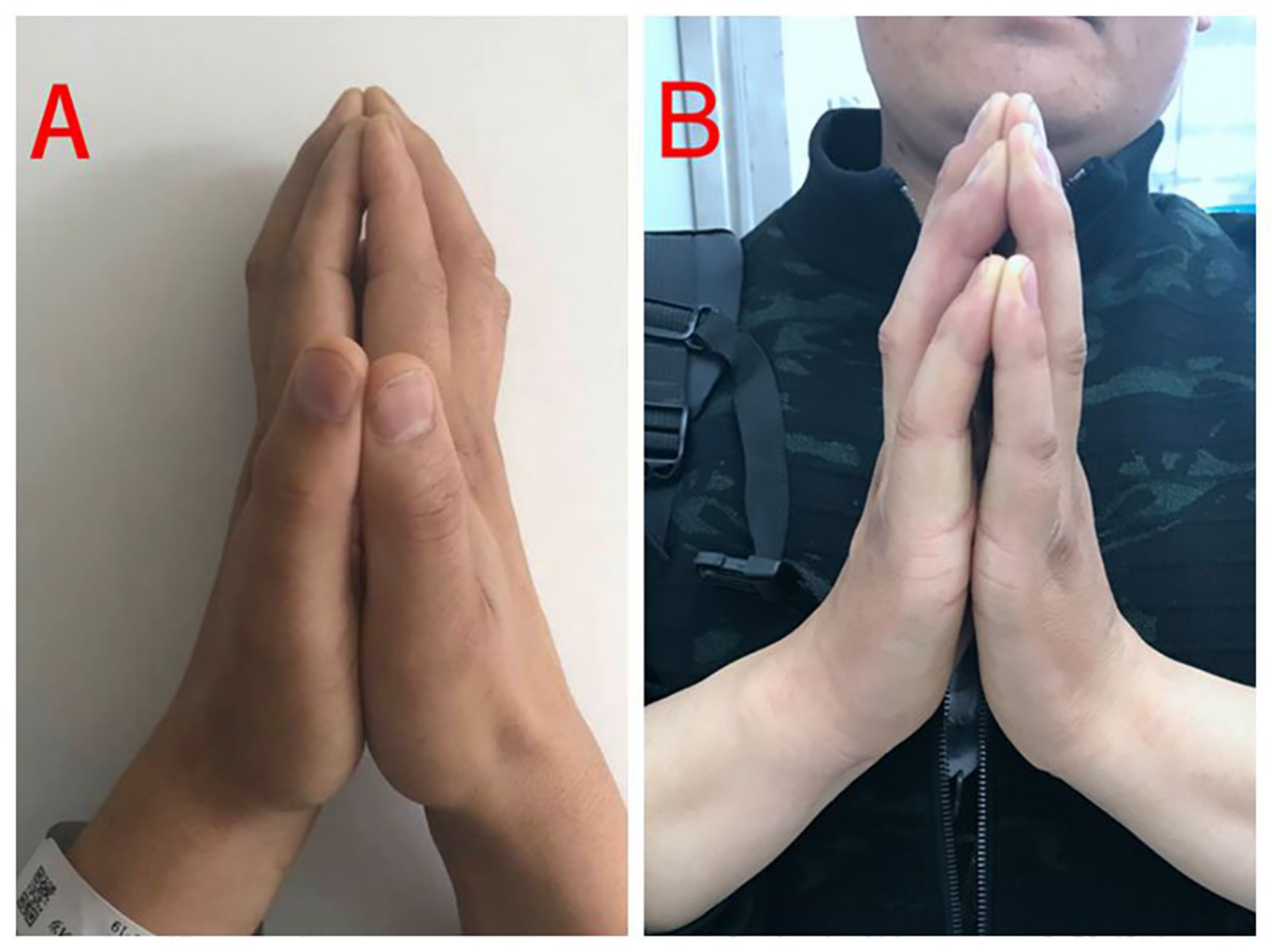
positive prayer sign (A) Before treatment. (B) Six months after treatment.

**Figure 2:**
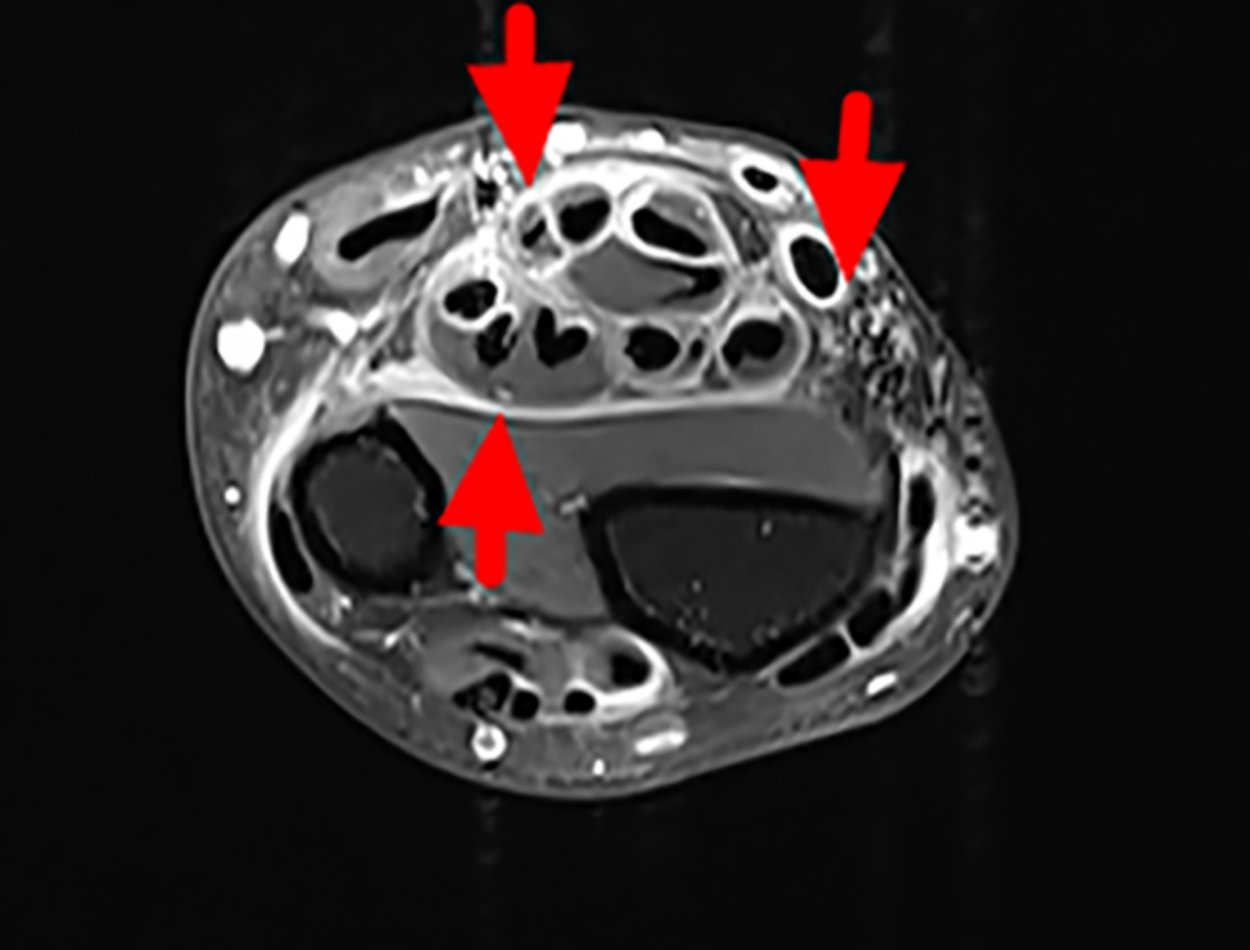
MR scan of the left wrist Axial enhanced T1-weighted fat-suppressed MR images showing thickening and enhancement of the fascia and tenosynovitis of flexor and extensor tendons. MR, magnetic resonance

On admission, physical examination showed vital signs normal, induration of both forearms around 10 centimeters above the wrists with normal superficial skin, groove sign on the back of both hands, limitation of dorsiflexion of the wrists, prayer sign positive (Figure 1A), and limitation of motion of both knees and right ankle. Laboratory findings were as follows: white blood cell count of 5.72 × 10^9^/L (normal range: 4.0-10.0), eosinophil absolute count of 0.76 × 10^9^/L (normal range: 0-0.3) and eosinophil percentage of 13.2%, hemoglobin of 120 g/L (normal range: 120-160), and platelet count of 314 × 10^9^/L (normal range: 100-300); urinalysis was normal. Both renal and liver function parameters were within the normal ranges. Erythrocyte sedimentation rate (ESR) was 54 mm/h (normal range: 0-20), C-reactive protein (CRP) was 25.1 mg/L (normal range: 1-8), immunoglobulin G of 18.3 g/L (normal range: 7.51-15.6), and C3 complement fraction of 1.27 g/L (normal range: 0.73-1.46); rheumatoid factor, human leukocyte antigen B27, antinuclear antibody, anticardiolipin antibody, and antineutrophil cytoplasmic antibody were all negative. Serum protein electrophoresis and immunofixation electrophoresis were normal. X-ray of the hands and knees showed no bone erosions. Computed tomography (CT) scan of the chest, abdomen, and pelvis showed no evidence of solid tumor. Single-photon emission CT (SPECT) of the bones was normal. Contrast-enhanced magnetic resonance imaging (MRI) scan of the left wrist showed thickening and enhancement of the muscular fascia and tenosynovitis of flexor and extensor tendons, without other pathological findings in hand joints (Figure 2). A full-thickness biopsy was planned; however, the patient and his family refused due to the fear of poor wound healing. According to the criteria proposed by Pinal-Fernandez et al. (Table 1), the diagnosis of EF was made [2].

**Table 1:** Proposed criteria for eosinophilic fasciitis

Major criteria
1. Swelling, induration, and thickening of the skin and subcutaneous tissue that is symmetrical or non-symmetrical, diffuse (extremities, trunk, and abdomen), or localized (extremities)
2. Fascial thickening with the accumulation of lymphocytes and macrophages with or without eosinophilic infiltration (determined by full-thickness wedge biopsy of clinically affected skin)
Minor criteria
1. Eosinophilia ＞0.5 × 10^9^/L
2. Hypergammaglobulinemia ＞1.5 g/L
3. Muscle weakness and/or elevated aldolase levels
4. Groove sign and/or peau d'orange appearance
5. Hyperintense fascia on T2-weighted magnetic resonance images
Exclusion criteria: diagnosis of systemic sclerosis; the presence of both major criteria, or one major criterion plus two minor criteria, establishes the diagnosis of eosinophilic fasciitis

The patient was treated with prednisone (1 mg/kg/day) and methotrexate (10 mg/kg/day) for a week. Two weeks later, the prednisone dose was tapered gradually, and a dose of 5 mg per day was maintained. The case was followed up for eight months; there was a significant improvement in subcutaneous tissue thickening and joint mobility (Figure 1B). ESR, CRP, and immunoglobulin G returned to normal.

## Discussion

EF is an uncommon disease that primarily affects adults between 40 and 50 years of age, with no sex bias, but it can be seen earlier in men. EF is characterized by inflammation, swelling, and thickening of the skin and fascia. The fascia is a sheet or band of fibrous connective tissue under the skin that separates different layers of tissues under the skin [3]. Its etiology remains unknown, but 30-60% of cases may have a recent history of strenuous exercise or trauma [4]. This is consistent with our patient since his work involved lifting heavy objects in the recent 10 years. Classically, EF starts with edema and erythema, and, subsequently, symmetrical induration of the extremities appears. The subcutaneous induration could lead to joint contractures and tendon retraction, which reflect the severity of fibrosis. Inflammatory arthritis can be seen in up to 40% of patients [4]. Visceral involvement is rare. Our case had oligoarthritis involving both knees and right ankle, and subcutaneous induration of both forearms, leading to a positive prayer sign. Laboratory tests revealed peripheral eosinophilia, hypergammaglobulinemia, and high inflammatory markers including ESR and CRP. Although fascial biopsy remains the gold standard for the diagnosis of EF, the role of MRI has been greatly emphasized in recent years, and diagnosing EF with MRI instead of biopsy has become a trend currently. MRI shows high signal intensity of myofascia on T2-weighted sequences or STIR (short tau inversion recovery), with enhancement after gadolinium injection on T1-weighted sequences [5-7]. Our patient’s MRI features are consistent with EF.

The subcutaneous induration could lead to joint contractures and tendon retraction, presenting as a prayer sign. Positive prayer sign is a rare and unspecific condition and can be seen in many other diseases besides EF. We searched PubMed for reports of cases by using the keywords “carpal tunnel syndrome” and “prayer sign”. Earlier reports have mentioned the presence of an associated carpal tunnel syndrome in around one-third of EF patients [8]. Involvement of the median nerve is detected clinically or by EMG (electromyography)/nerve conduction study. Its underlying pathophysiological mechanism appears to be fibrosis secondary to inflammation of the tenosynovium of the flexor tendons and compression of the median nerve in the limited space of the carpal canal. There have been only two EF cases involving the wrists with a positive prayer sign reported in the literature in the recent 10 years, of which the clinical manifestations are similar to those of our patient [9-10]. Other diseases including diabetes mellitus, amyloidosis, and paraneoplastic syndrome can also cause a positive prayer sign due to the thickening of the dermis and the fibrosis of the subcutaneous tissue in the wrists [11-13]. These diseases could be ruled out based on our laboratory and imaging investigations. In addition, EF should also be differentiated from systemic sclerosis (SSc). The typical features of SSc, such as Raynaud's phenomenon, sclerodactyly, microstomia, and telangiectasia, were not observed in our patient. Consequently, according to the criteria proposed by Pinal-Fernandez et al., the diagnosis of EF was made [2].

In regard to therapy, data from retrospective studies increasingly favor the combination of systemic corticosteroids (prednisone 0.5-1 mg/kg/day) and methotrexate (10-25 mg once weekly) as the initial treatment [14]. A study showed that patients had a response rate of more than 70% to oral prednisone [15]. In a report by Lebeaux et al., 15 out of 32 patients were treated with steroid pulse therapy. The rate of complete remission was higher in the treated group than the non-treated group (87% vs. 53%; P = 0.06) [16]. There are a comparatively large number of reports on methotrexate. Berianu et al. treated 16 patients with methotrexate and reported that 3 patients had complete remission and 7 had partial remission [17]. In a new retrospective study of 89 patients, methotrexate was the most commonly used immunosuppressant in 79%, hydroxychloroquine in 45%, mycophenolate mofetil in 18%, and azathioprine in 8%. The response rate was similar for a single immunosuppressant agent [18]. The therapeutic response and prognosis are generally favorable. In the follow-up duration of eight months, our patient showed good response to the treatment of prednisone and methotrexate.

## Conclusions

Taken together, it is important to emphasize that a positive prayer sign or carpal tunnel syndrome might be the first symptom of EF. However, other systemic inflammatory and infiltrative disorders that thicken the transverse carpal ligament or decrease the space available for the median nerve in the carpal canal should not be ruled out. Although a full-thickness biopsy is the gold standard for the diagnosis of EF, contrast-enhanced MRI scan has become of great significance, as MRI can detect the inflammation and thickening of the fascia. The combination regimen of systemic corticosteroids and methotrexate is recommended.
